# Changes in Natural Killer Cell Activation and Function during Primary HIV-1 Infection

**DOI:** 10.1371/journal.pone.0053251

**Published:** 2013-01-09

**Authors:** Vivek Naranbhai, Marcus Altfeld, Salim S. Abdool Karim, Thumbi Ndung'u, Quarraisha Abdool Karim, William H. Carr

**Affiliations:** 1 CAPRISA – Centre for the AIDS Programme of Research in South Africa, Doris Duke Medical Research Institute, Nelson R Mandela School of Medicine, University of KwaZulu-Natal, Durban, South Africa; 2 HIV Pathogenesis Programme, Doris Duke Medical Research Institute, Nelson R Mandela School of Medicine, University of KwaZulu-Natal, Durban, South Africa; 3 Ragon Institute of Massachusetts General Hospital, Massachusetts Institute of Technology and Harvard, Boston, Massachusetts, United States of America; 4 Department of Epidemiology, Mailman School of Public Health, Columbia University, New York, New York, United States of America; Emory University School of Medicine, United States of America

## Abstract

**Background:**

Recent reports suggest that Natural Killer (NK) cells may modulate pathogenesis of primary HIV-1 infection. However, HIV dysregulates NK-cell responses. We dissected this bi-directional relationship to understand how HIV impacts NK-cell responses during primary HIV-1 infection.

**Methodology/Principal Findings:**

Paired samples from 41 high-risk, initially HIV-uninfected CAPRISA004 participants were analysed prior to HIV acquisition, and during viraemic primary HIV-1 infection. At the time of sampling post-infection five women were seronegative, 11 women were serodiscordant, and 25 women were seropositive by HIV-1 rapid immunoassay. Flow cytometry was used to measure NK and T-cell activation, NK-cell receptor expression, cytotoxic and cytokine-secretory functions, and trafficking marker expression (CCR7, α_4_β_7_). Non-parametric statistical tests were used. Both NK cells and T-cells were significantly activated following HIV acquisition (p = 0.03 and p<0.0001, respectively), but correlation between NK-cell and T-cell activation was uncoupled following infection (pre-infection r = 0.68;p<0.0001; post-infection, during primary infection r = 0.074;p = 0.09). Nonetheless, during primary infection NK-cell and T-cell activation correlated with HIV viral load (r = 0.32'p = 0.04 and r = 0.35;p = 0.02, respectively). The frequency of Killer Immunoglobulin-like Receptor-expressing (KIR_pos_) NK cells increased following HIV acquisition (p = 0.006), and KIR_pos_ NK cells were less activated than KIR_neg_ NK cells amongst individuals sampled while seronegative or serodiscordant (p = 0.001;p<0.0001 respectively). During HIV-1 infection, cytotoxic NK cell responses evaluated after IL-2 stimulation alone, or after co-culture with 721 cells, were impaired (p = 0.006 and p = 0.002, respectively). However, NK-cell IFN-y secretory function was not significantly altered. The frequency of CCR7+ NK cells was elevated during primary infection, particularly at early time-points (p<0.0001).

**Conclusions/Significance:**

Analyses of immune cells before and after HIV infection revealed an increase in both NK-cell activation and KIR expression, but reduced cytotoxicity during acute infection. The increase in frequency of NK cells able to traffic to lymph nodes following HIV infection suggests that these cells may play a role in events in secondary lymphoid tissue.

## Introduction

Understanding immunological responses that modulate HIV-1 pathogenesis is important for vaccine and immunotherapy development. Events that occur during the earliest period of HIV-1 infection disproportionately influence the outcome and course of disease. In particular, generalized activation of CD8 T-cells is associated with faster disease progression [1], whilst HIV-specific CD8+ and CD4+ T-cell responses during primary infection are associated with slower disease progression and lower set point viral load [2], [3], [4]. Although the effects of HIV-1 infection on adaptive immune cells, particularly T-cells have been well described, the impact on innate immune responses are less well understood. Natural Killer (NK) cells, are part of the innate immune defense against viral infections and modulate subsequent adaptive immune responses [5]. *In vitro* and animal studies suggest a possible role of NK cells in controlling viral replication during primary HIV-1 infection [6]. NK cells can limit HIV replication through direct killing of infected cells as well as the secretion of anti-viral cytokines. However, HIV can also impair immune responses by NK cells [7], [8], [9]. By examining the responses of NK cells prior to infection and at the earliest time points following infection, we may better unravel cause-effect relationships of HIV impact on immune responses and vice-versa.

During chronic HIV-1 infection NK-cell cytotoxicity and cytokine secretion are impaired, but these deficiencies likely start earlier in the course of disease [10]. The impairments are associated with expansion of an “anergic” NK cell subset that expresses CD16 and relatively low levels of CD56 (CD56_neg_CD16_pos_). Some investigators have also proposed that this subset functionally impairs the total NK cell population [11]. *In vitro* models of HIV infection suggest that HIV viraemia contributes to this impairment [9]. Viraemia peaks early following acquisition, during primary HIV infection. Thus, NK cell dysregulation *in vivo* likely begins during primary infection. This hypothesis is supported by the findings of Alter and colleagues [7]. In a study of 10 acutely infected individuals, they reported quantitative expansion of NK cell populations during the seronegative phase of primary HIV-1 infection that rapidly returned to baseline, but remained qualitatively different from 14 healthy HIV-uninfected individuals, or 45 individuals with chronic HIV. They observed NK cell expansion prior to the development of adaptive (CD8+ T-cell) responses. They also found that NK-cell degranulation and interferon-gamma (IFN-γ) secretion were elevated in the seronegative phase of infection but declined subsequently independent of antiretroviral treatment status. However the lack of blood samples from the same individuals prior to HIV acquisition limited the conclusions from this study [12].

Few studies have quantified immune responses before and after HIV infection among the same individuals. Most previous studies compare immune responses in HIV acutely infected individuals to uninfected individuals [13]. Here we extend the findings of previous studies by characterizing innate immune responses in the same individuals before and after HIV acquisition. To better understand the kinetics of NK-cell responses in primary HIV-1 infection we compared innate immune responses in paired blood specimens collected prior to and during primary infection among women who acquired HIV whilst enrolled in a randomized, controlled clinical trial of 1% Tenofovir microbicide gel. Our findings provide new insights into the effects of HIV on NK-cell responses.

## Methods

### Study Subjects

This prospective cohort study was nested in the CAPRISA 004 Tenofovir gel trial of coitally-related use of 1% Tenofovir gel [14]. The trial enrolled 889 sexually active (defined as >2 sex acts in preceding 30 days) women 18–40 years of age, who were resident in high-prevalence rural and urban communities. For this study, 41 consenting adult women, who acquired HIV whilst in the trial, were selected based on sample availability. Incident HIV infection was established by two rapid HIV antibody tests and confirmed by negative qualitative HIV PCR testing of pre-infection samples. In cases of diagnostic uncertainty, Western blots and/or an HIV-1 ELISA were performed. To characterize changes in NK cells in women who acquired HIV-1, and whether these were associated with changes in T-cell activation or function, we examined NK cell and T-cell responses at the last sampling time point before infection and during primary HIV-1 infection.

Participants gave informed consent for their samples to be stored and used for this study. This study was approved by the University of KwaZulu-Natal Biomedical Research Ethics Committee (#BE073/010). The clinical trials registration number of parent trial was NCT00441298.

### Natural Killer cell and T-cell activation

Activation, CD107a expression and intracellular cytokine production were measured in cryopreserved peripheral blood mononuclear cells (PBMC) in batch analyses using optimized flow cytometry methods as previously described [15]. Cryopreserved PBMC were rapidly thawed in warm media (RPMI 1640; Gibco), washed and rested for two hours. Sample viability and cell counts were determined by staining with Viacount (Millipore) and run on a Guava PCA (Millipore). PBMC were stained with Yellow Viability Dye (Invitrogen), anti-CD14 (clone Tuk4, Invitrogen) and -CD20 (clone HI47, Invitrogen) antibodies (to exclude nonviable cells, monocytes and B-cells, respectively). In addition, the cells were stained with anti-CD3 (clone SP34-2, BD Biosciences),-CD16 (clone 3G8, BD Biosciences),-CD38 (clone HIT2, BD Biosciences),-CD56 (clone B159, BD Biosciences),-CD69 (clone FN50, BD Biosciences),-CD158a (cone HP-3E4, BD Biosciences),-CD158b (clone CH-L, BD Biosciences), -HLA-DR (clone L243, BD Biosciences) and anti-CD158e1/e2 (clone Z27, Beckman Coulter) antibodies. Flow cytometry data were collected on a BD LSRII and analyzed using FlowJo v9.3.1 (Treestar). NK cells were defined as viable, CD14_neg_, CD20_neg_, CD3_neg_, CD16/56_pos_ lymphocytes. The frequency of NK cells that were HLA-DR_pos_ was determined using fluorescence minus one (FMO) gates. The frequency of T-cells, defined as viable, CD14_neg_, CD20_neg_ CD3_pos_ cells, expressing both CD38 and HLA-DR, was similarly determined. The gating strategy is shown in Figure S1.

### NK-cell effector functions

Using cryopreserved PBMCs in batched analyses each of the following three NK-cell effector functions were quantified following IL-2 stimulation (100 U/ml R+D Systems) alone, IL-2 plus 721 cells or PMA/Ionomycin: degranulation (using CD107a expression as a surrogate [16]), cytokine secretion (IFN-γ), and recent cell division (using Ki-67 as a surrogate [17]). Cryopreserved PBMC were rapidly thawed in warm media, washed and rested for 12 hours in IL-2 enriched culture medium (RPMI 1640 media containing 50 U/ml IL-2 and 10% fetal calf serum). For experiments in which cells were stimulated with 721 cells, the NK cells were cultured with equal numbers of 721 cells. Cells were washed, resuspended in culture medium containing an antibody directed against CD107a, and cultured for a further 6 hours. Samples were processed for flow cytometry as detailed above with the following modification. Cells were surface stained with anti-CD3 (clone SP34-2, BD Biosciences), -CD8 (clone 3B5, Invitrogen), -CD56 (clone B159, BD Biosciences), -CD69 (clone FN50, BD Biosciences) and anti-CD7 (clone 8H8.2, Beckman Coulter) antibodies, then washed, fixed and permeabilised before staining with anti-Ki-67 (clone B56, BD Biosciences) and -IFN-γ (clone 4S.B3, BD Biosciences) antibodies, respectively. To account for NK cells that have lost CD16 expression due to activation and to exclude monocyte/DC-like cells, anti-CD7 antibody was used as described [18]. FMO gates were used to determine the proportion of cells responding with one or more effector functions (i.e., CD107a, IFN-γ or Ki-67). The gating strategy is shown in Figure S2.

### Statistical methods

Assays were conducted blinded to time point of sampling. For comparisons between paired specimens at pre- and post-infection time points from the same individual, a non-parametric Wilcoxon signed rank test was performed. For comparisons between different time points post-infection, a Kruskal-Wallis test was performed. Statistical analyses were conducted in GraphPad Prism v5 (GraphPad).

## Results

To characterize the earliest changes in innate and adaptive immune responses induced by HIV-1, we examined NK-cell and T-cell responses, respectively, prior to HIV acquisition and during primary infection. Unlike most prior studies these responses were measured in the same individuals over time.

### Study cohort

A total of 41 recently infected women were included in this study, (mean age 23.3 years). Thirty-one were in the tenofovir arm and eight in the placebo arm. To take into account differences between post-infection sampling time points in this cohort, the results were stratified by stage of HIV-1 infection at the time of sample collection. Blood samples collected at the last visit prior to HIV acquisition (hereafter referred to as pre-infection) were collected at a median of 119 days prior to HIV acquisition, IQR 67.25–169.25). The samples collected from these women at the first visit following HIV infection were designated as post-infection samples. HIV serostatus (seronegative versus seropositive) of the samples was only used in reference to samples collected after HIV acquisition (i.e., post-infection). Five participants were sampled during the seronegative stage of primary infection based on two HIV rapid tests (*seronegative* group), 11 whilst having indeterminate or discordant antibody responses (*discordant* group) and 25 during the seropositive stage of infection (seropositive group). Due to limited cell numbers, we were unable to assess NK-cell degranulation, proliferation or cytokine secretion in two seronegative, two discordant, and three seropositive individuals.

Based on estimates of the date of infection confirmed by BEAST analysis of viral sequences, seronegative, discordant and seropositive individuals were sampled at a median (IQR) of 14 (13.5–14), 15 (13–25) and 131 (105.5–157) days post-infection respectively. The median viral load (log_10_ copies/ml) (IQR) was 4.95 (4.39–5.29), 4.63 (4.19–5.34) and 3.86 (3.38–4.80) for seronegative, discordant and seropositive groups respectively. The corresponding probable Fiebig staging was based on a combination of the estimated time post-infection at sampling and ancillary HIV PCR, Western blot and/or ELISA results, where available, were I/II/early III; III/IV, and V/VI for seronegative, discordant and seropositive groups, respectively.

Consistent with a previous report on this cohort by Mureithi et al. [19] we did not observe any significant differences in any of the measured parameters between women who acquire HIV whilst receiving 1% Tenofovir gel compared with women on placebo gel. For this reason the groups were combined in further analyses.

### NK-cell and T-cell activation were uncoupled by primary HIV-1 infection, but both were positively correlated with HIV viral load

**Figure 1:**
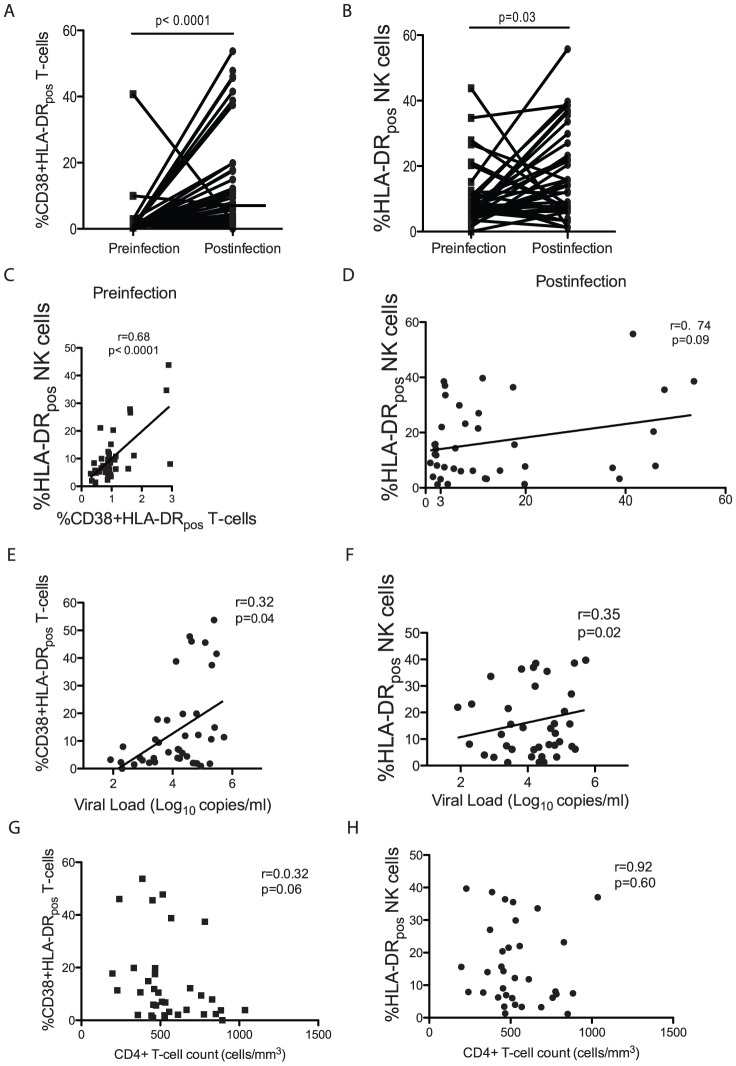
Primary HIV-1 infection was associated with differential T-cell and NK-cell activation. T-cells (A) and NK cells (B) were activated following HIV infection. The proportion of activated T-cells and NK cells was significantly correlated before (C), but not following HIV-1 infection (D). In contrast, during primary infection, both T-cell (E) and NK-cell (F) activation were positively correlated with HIV viral load (log_10_ copies/ml) but not CD4+ T-cell counts (G and H respectively).

The proportions of activated T-cells and NK cells were significantly increased during primary HIV-1 infection (p<0.0001 and p = 0.03, Figures 1A and 1B, respectively). Prior to HIV acquisition, the proportions of activated NK cells and T-cells were positively correlated (r = 0.68, p<0.0001, Figure 1C), whereas following infection, they were uncoupled (r = 0.074, p = 0.09, Figure 1D). Nevertheless, the proportions of activated T-cells and NK cells correlated independently with HIV viral load (r = 0.32, p = 0.04 and r = 0.35, p = 0.02, respectively; Figures 1E and 1F) but not with CD4+ T-cell counts (Figure 1G and H).

As a proportion of lymphocytes, NK cells were not significantly expanded during primary infection (Figure S3A). To take into account differences between post-infection sampling time points in this cohort the results were stratified by stage of HIV-1 infection when samples were collected. We observed no significant difference in frequencies of NK cells during primary infection between seronegative, serodiscordant and seropositive individuals (p = 0.41; Figure S3B) although a weak trend to greater frequencies in seronegative individuals was present. Similarly, there was no difference in the frequencies of recently divided NK cells as measured by expression of Ki-67 (Figure S3C and 3D). Although the difference in proportions of activated NK cells was not statistically significant, there was a tendency for greater activation during later stages of primary infection (Figure S3E). The frequencies of the CD56_neg_ (anergic), CD56_dim_ and CD56^hi^ NK cell subsets did not differ between pre-infection and post-infection time points, nor amongst the stages of primary infection (Figure S4A and 4B). In contrast, the proportion of recently divided CD8+ T-cells increased following HIV-1 infection (p<0.0001, Figure S5A).

### The frequency of Killer Immunoglobulin-like Receptor expressing NK cells (KIR_pos_) increased following HIV-1 infection with KIR_pos_ NK cells being less activated than KIR_neg_ NK cells

**Figure 2:**
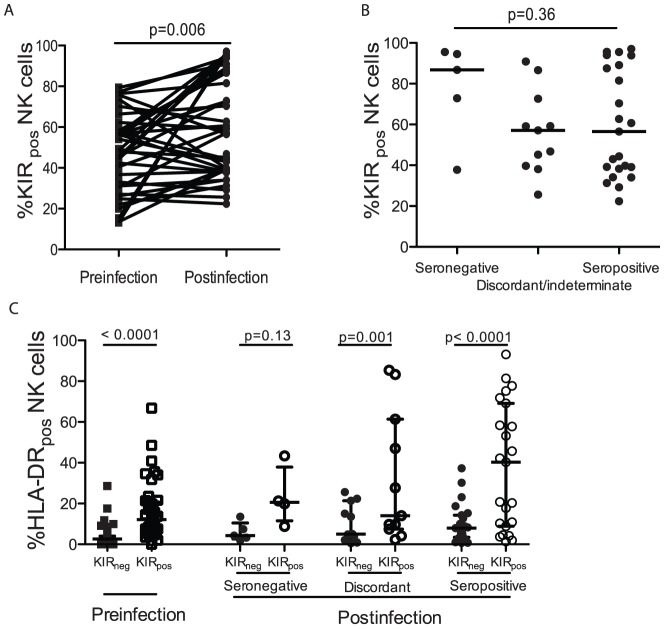
Following infection, an increased proportion of activated NK cells in blood expressed Killer Immunoglobulin-like Receptors (KIR). The proportion of KIR_pos_ NK cells in blood increased following HIV infection (A). The proportion of KIR_pos_ NK cells did not differ between stages of primary HIV infection (seronegative, sero-discordant or seropositive stages) (B). KIR_pos_ NK cells were more activated than KIR_neg_ NK cells (C).

Next, to explore whether KIRs contributed to the observed changes in activation profiles of NK cells, KIR expression was evaluated by flow cytometry. For this analysis commercial antibodies, which detected the most commonly expressed KIRs (KIR2DL1, KIR2DS1, KIR2DL2, KIR2DL3, KIR3DL1 and KIR3DS1), were used. A significant expansion in the proportion of KIR_pos_ NK cells was evident following HIV-1 infection (p = 0.006, Figure 2A) and appeared higher in individuals with seronegative primary HIV-1 infection but this was not significant (Figure 2B). Since KIR modulate NK cell activation, activation on KIR_pos_ and KIR_neg_ cells was compared in each category of samples. Prior to HIV infection, the proportion of KIR_pos_ NK cells that were activated was significantly lower than KIR_neg_ NK cells (p<0.0001, Figure 2C). During primary HIV-1 infection, KIR_pos_ NK cells were similarly less activated than KIR_neg_ NK cells amongst those with discordant or seropositive primary HIV-1 infection (p = 0.001, p<0.0001 respectively, Figure 2C). This did not achieve statistical significance in the smaller group with seronegative primary HIV-1 infection (p = 0.13, Figure 2C).

### NK cell function was impaired during primary HIV-1 infection

To quantify the effects of HIV on NK-cell function, we assessed NK cell degranulation and interferon-gamma (IFN-γ) secretion in paired blood samples collected prior to HIV acquisition and after infection, during the early stages of primary infection. The samples were analyzed after *ex vivo* culture in media containing Interleukin-2 (IL-2) alone, or after exposure to 721 cells (a B-cell line deficient in HLA class 1 expression) and IL-2, or PMA/Ionomycin and IL-2. For this assay NK cells were defined as CD3_neg_ cells expressing CD7, as done previously by Milush and colleagues [18].

**Figure 3:**
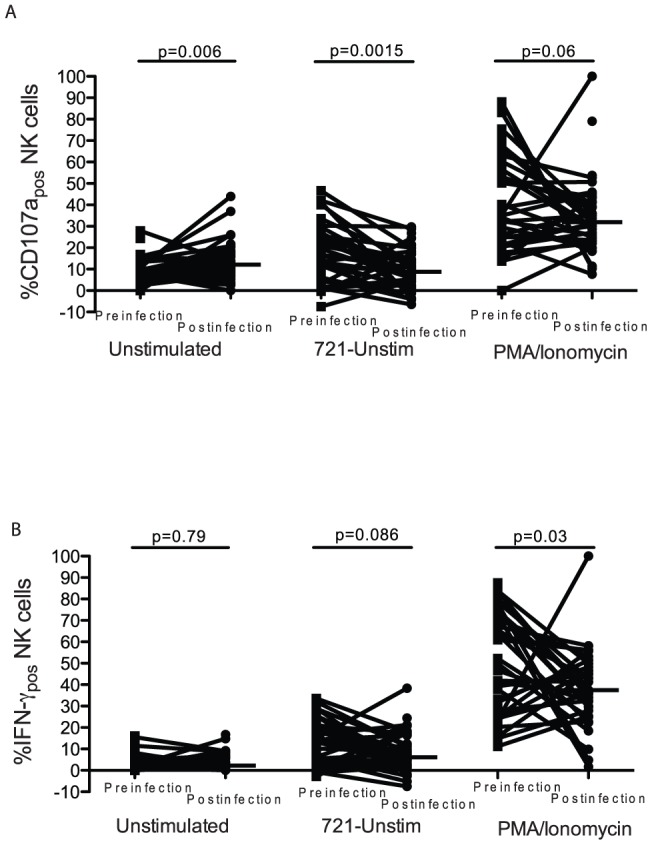
Following HIV infection NK-cell responses to stimulation were diminished. Natural Killer cell degranulation (A) and IFN-γ secretion (B) responses after stimulation with IL-2 alone or with IL-2 and 721 cells (adjusted for background) or with PMA/Ionomycin. Data are adjusted for background responses to media alone.

Following *ex vivo* culture in media containing IL-2 stimulation alone, the proportion of degranulating NK cells was elevated during primary HIV-1 infection compared to prior to HIV acquisition, (p = 0.006, Figure 3A). In contrast, the degranulation response of NK cells to 721 cells, (a model of “missing self”) [20], was significantly reduced during primary HIV-1 infection (p = 0.002, Figure 3A). Similarly, stimulation by PMA/Ionomycin showed the same tendency, but this difference did not reach statistical significance (p = 0.06, Figure 3A). There was no difference in the proportion of NK cells secreting IFN-γ following *ex vivo* IL-2 stimulation alone (Figure 3B). Following stimulation with 721 cells there was a tendency of fewer NK cells secreting IFN-γ after HIV acquisition (p = 0.09, Figure 3B). Likewise, the proportion of NK cells secreting IFN-γ following stimulation with PMA/Ionomycin was significantly lower after infection (p = 0.03). In contrast to NK cells, the proportion of degranulating CD8+ T was increased following HIV-1 infection (p = 0.02, Figure S5B) and there was no change in the proportion of IFN- γ_pos_ CD8+ T-cells (Figure S5C).

### The frequency of NK cells expressing markers for homing to lymphoid tissues is increased during primary HIV-1 infection

**Figure 4:**
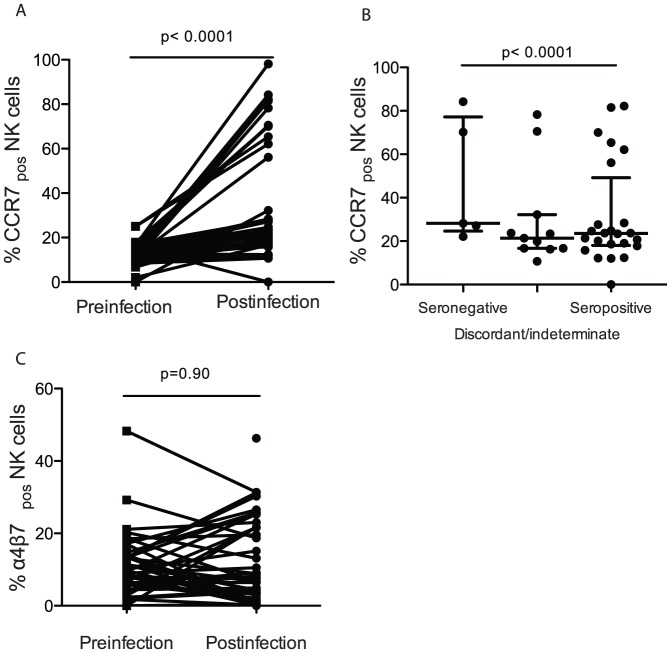
Early after HIV infection blood NK cells increased their expression of lymphoid, but not gut homing receptors. The proportion of NK cells expressing CCR7 was increased following HIV infection (A). The proportion of NK cells expressing CCR7 was reduced during later stages of primary infection (B). The proportion of NK cells that expressed α4β7, was not changed during primary HIV-1 infection (C).

Finally, to measure the impact of HIV acquisition on NK cell trafficking we quantified the expression of receptors for lymphoid and gastrointestinal tissue homing. We quantified the frequency of NK cells expressing either CCR7, which enables trafficking to lymphoid tissues [21], or α_4_ and β_7_ integrins, which enable homing to the gut [22]. After infection the frequency of CCR7+ cells increased markedly (p = <0.0001, Figure 4A). The proportion of CCR7+ NK cells was decreased with later category of primary HIV-1 infection but did not return to baseline (p<0.0001, Figure 4A). In contrast, the proportion of NK cells expressing both the α_4_ and β_7_ integrin subunits remained unchanged (Figure 4B).

## Discussion

Primary HIV-1 infection is associated with differential NK cell and T-cell activation, expansion of NK cells expressing KIR, impairment of cytotoxic NK cell function in the absence of significant increase in anergic subsets, and NK cells equipped to home to secondary lymphoid tissues. These data offer insight into the impact of primary HIV-1 infection on NK cells.

Unlike prior studies that cross-sectionally analysed acutely infected and uninfected individuals [7], [12], this study was unique in following immune responses over-time before and after HIV acquisition. The relatively small sample size of samples collected during the seronegative phase of infection limited our ability to compare responses during the seonegative versus seropositive stages of acute infection. Nevertheless, our finding that both NK cells and T-cells were activated early following HIV infection was consistent with previous findings by Alter and colleagues [12]. However, during primary infection we did not observe a significant expansion in the frequency of NK cells amongst total lymphocytes that has been described previously [12]. Alter and colleagues noted an expansion of NK cells among HIV-infected, seronegative individuals compared to uninfected individuals. Since most of the post-infection samples used in this study were collected during the seropositive phase of primary infection, we speculate that NK-cell expansion may have occurred prior to our sampling time points. In addition, the relatively low number of seronegative individuals limited our statistical power to detect these differences. Therefore, our results are consistent with other reports that suggest that NK cell population expansion occurs early in primary infection but may be transient [7], [12].

In this study NK cell and T-cell activation were uncoupled by primary HIV-1 infection illustrating the differential effects of HIV on NK cells and T-cells. This finding appears to be mostly explained by the relatively greater increase in T-cell activation. Alter and colleagues have previously shown that T-cell responses occur after NK-cell responses and are inversely correlated with the magnitude of NK cell activity during primary infection [23]. Primary HIV-1 infection was associated with expansion of KIR_pos_ NK cells, consistent with a previous study, but was not associated with enhanced KIR_neg_ NK cell activation. We speculate that this may be due to either expansion of NK cells able to detect HIV-1-infected cells that down-regulate HLA expression, or a regulatory response that diminishes NK cell reactivity. Fogli and colleagues have previously demonstrated that KIR_pos_ NK cells are incompletely activated in individuals with chronic HIV infection [8], which implies that this may be a regulatory mechanism. In this study we were not able to delineate which specific KIR (inhibitory versus activating) were expressed on the surface of NK cells. Further studies using newer reagents that can distinguish specific KIR are needed to understand the regulation of KIR expression in NK cells during primary infection.

Consistent with previous studies, NK cell function following stimulation with 721 cells or PMA/Ionomycin was impaired as early as primary HIV-1 infection even in the absence of significant expansion of anergic NK cells (CD56_neg_CD16_pos_) [7]. After infection, a higher proportion of NK cells degranulated in the presence of only IL-2 than before infection. This suggests that HIV may prime responses or that NK cells are degranulating *in vivo* in response to HIV. However, their response to culture with 721 cells was diminished. Similarly, their responses to non-specific stimulation with PMA/Ionomycin were reduced. These results suggest that NK cell functional deficiencies during primary infection might be attributed to impaired or incomplete activation rather than the accumulation of anergic NK cells.

Finally, we observed an increase in the frequency of blood NK cells expressing CCR7, a receptor for homing to lymphoid tissues. We did not observe a similar expansion of NK cells expressing α_4_β_7_ integrins for homing to the gut. In contrast to our findings, Luteijn and colleagues did not observe a higher frequency of CCR7+ NK cells in blood from HIV-infected compared to uninfected individuals in their cross-sectional analysis [24]. One difference between these studies is that the participants in the study by Luteijn et al. were beyond the acute phase, but within the first year of HIV infection. Thus, the changes in CCR7 expression that we observed may be transient and limited to the acute phase of infection. In a previous study we found that chemokine receptor expression on NK cells changes rapidly [15]. In addition, the wide variation between individuals in the magnitude of change may also explain differences in the outcomes. In this study we noted a relatively large increase in the proportion of CCR7+ NK cells among some individuals and a much more subtle increase among others. Longitudinal assessment of changes in the frequency of CCR7+ NK cells within individuals would take some of this variability into account, and thus enable the detection of differences not apparent in a cross-sectional analysis. We propose that the increase in the frequency of KIR_pos_ NK cells in blood may also increase the proportion of NK cells that are able to traffic to lymphoid tissues. In support of this model, Marcenaro and colleagues previously showed that KIR-ligand mismatch was required for the *ex vivo* up-regulation of CCR7 expression by human NK cells interacting with either monocyte-derived dendritic cells or Epstein Barr Virus (EBV)-transformed B-cell lines [25]. It has been well-described that HIV decreases the expression of selected HLAs, which are ligands for KIRs [26]. Based on these observations, we speculate that the expansion of KIR-expressing NK cells concurrent with relative reduction in HLA-ligand expression simulates KIR-ligand mismatch and may facilitate NK cell CCR7 up-regulation and homing to lymphoid tissues. But Luteijin and colleagues observed relatively few KIR_pos_ NK cells in the lymph nodes of HIV-infected individuals [24] suggesting that these events may be transitory. Additional studies are need to determine if changes in NK-cell compartmentalization occur *in vivo* and whether it influences the viral load set point in the acute phase of HIV infection. Studies may also need to track NK cell homing longitudinally in order to determine whether, for example, the observation of lack of α_4_β_7pos_ NK cell expansion is because NK cells home to the gut earlier than we were able to observe.

Several factors limit our conclusions. Firstly, we previously found that women in this cohort, who acquired HIV-1, had higher activation and lower NK cell responses prior to infection than women who remained uninfected [27], [28]. Hence, these results may be generalizable to South African women who subsequently acquire HIV heterosexually, but they may not be generalizable to other populations. Future studies should consider whether pre-infection immune responses confound inferences about immune responses in the early post-infection period. Secondly, most women in this study were studied after the earliest phase of primary HIV-1 infection, as most had either discordant or positive serology at the time of first sampling. Thus transient events that occur very early infection may have been missed.

Overall, we found that primary HIV-1 infection was associated with activation of NK cells, potentially modulated by KIR expression. We observed functional impairment of NK cells early after HIV acquisition in the absence of anergic NK cell expansion. Further, during primary infection NK cells acquired the ability to home to secondary lymphoid tissues. This implies that they may play a role in early events outside the peripheral blood compartment. This study of matched blood specimens obtained before HIV-1 acquisition and during the stages of primary infection has helped delineate directionality in the relationship between NK cells and HIV during primary HIV-1 infection.

## Supporting Information

Figure S1 The gating strategy for defining NK cells that expressed HLA-DR, a marker of activation, in matched samples obtained pre-infection (A) or post-infection (B), included (clockwise from top left for A and B) the gating of singlets, with forward (FSC) and side-scatter (SSC) properties consistent with lymphocytes, that were not monocytes (CD14_neg_), B-cells (CD20_neg_) or dead cells (Viability dye_neg_) and that were CD3 negative and expressed CD16/CD56. Figure shows a single donor at each timepoint.
(EPS)

Figure S2 The gating strategy for defining NK cells expressing CD107a or IFN- γ and obtained pre-infection (A) or post-infection (B) were evaluated by multiparametric flow cytometry. NK cells were defined (shown clockwise for A and B) after gating on singlets, lymphocytes, non monocytes (CD14_neg_), non B-cells (CD20_neg_) or dead cells (Viability dye_neg_) and that were CD3 negative but expressed CD7 (as described [18]). For each donor, both pre-infection and post-infection responses were evaluated following stimulation with media containing rhIL-2 alone, or with 721 cells or PMA/Ionomycin.
(EPS)

Figure S3 The frequency of NK cells (A), and the frequency of recently divided NK cells (Ki-67_pos_, C) was not altered by HIV infection (B) nor during the stages of primary HIV infection (D). The proportion of activated NK cells was not altered during primary infection (E).
(EPS)

Figure S4 The proportion of anergic (CD56_neg_), cytotoxic (CD56_dim_) or cytokine-secreting (CD56_hi_) NK cells was not significantly altered by HIV infection (A) nor by stage of primary infection (B).
(EPS)

Figure S5 The proportion of recently divided CD8+ T-cells (A), and degranulating CD8+ T-cells (B) was increased in primary HIV infection but the proportion of CD8_pos_ T-cells secreting IFN-γ was not significantly altered (C).
(EPS)
